# Highly Efficient Multi Channel Packet Forwarding with Round Robin Intermittent Periodic Transmit for Multihop Wireless Backhaul Networks

**DOI:** 10.3390/s17112609

**Published:** 2017-11-13

**Authors:** Kazuki Maruta, Hiroshi Furukawa

**Affiliations:** 1 Graduate School of Engineering, Chiba University, 1-33 Yayoi-cho, Inage-ku, Chiba 263-8522, Japan; 2 Graduate School of Information Science and Electrical Engineering, Kyushu University, 744 Motooka, Nishi-ku, Fukuoka 819-0395, Japan; furuhiro@ait.kyushu-u.ac.jp

**Keywords:** wireless backhaul, multi-hop, intermittent periodic transmit, round robin, multi-channel, CSMA/CA

## Abstract

Round Robin based Intermittent Periodic Transmit (RR-IPT) has been proposed which achieves highly efficient multi-hop relays in multi-hop wireless backhaul networks (MWBN) where relay nodes are 2-dimensionally deployed. This paper newly investigates multi-channel packet scheduling and forwarding scheme for RR-IPT. Downlink traffic is forwarded by RR-IPT via one of the channels, while uplink traffic and part of downlink are accommodated in the other channel. By comparing IPT and carrier sense multiple access with collision avoidance (CSMA/CA) for uplink/downlink packet forwarding channel, IPT is more effective in reducing packet loss rate whereas CSMA/CA is better in terms of system throughput and packet delay improvement.

## 1. Introduction

Wireless infrastructure is now an indispensable means of communication for us. Mobile data traffic has been significantly increasing every year, not only the amount of data per unit time such as movie contents, but also the number of terminals has been increasing significantly [1]. The reason for this is that the concept of communication between devices such as internet of things (IoT) [2,3,4,5] and machine type communication (MTC) [6,7,8] has become widespread. In a sensor network that collects environmental information, wireless nodes are deployed in a wide area and in large quantities. On the other hand, the requirement for high capacity remained unchanged. Shrinking cell size is the most promising and straightforward approach to efficiently accommodate explosively increasing traffic by easing network load per base station (BS) [9,10,11]. The challenge of this approach is the efficient deployment of a large number of small cell BSs. It is extremely valuable to be able to provide a wireless access system that can satisfy both demands for higher data rate and massive traffic delivery in an efficient way.

**Figure 1:**
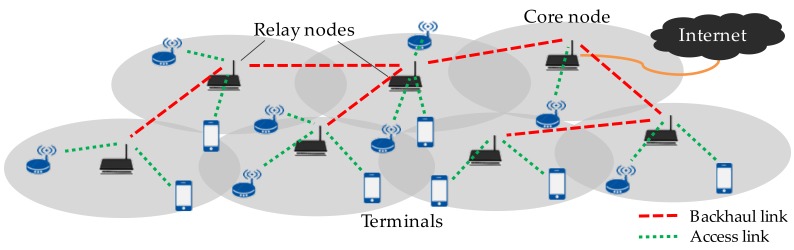
Multi-hop wireless backhaul network.

Multi-hop wireless backhaul networks (MWBN) [12,13,14] is known to be one of the most valuable means to support small cell deployment. Relay-based backhauling is expected to be further effective in the practical aspect for cost reduction [15,16]. As shown in Figure 1, access points compose a network via wireless connections and some of them are connected to outside of the network (i.e., Internet). Multi-hop relay can easily enhance the area coverage without deploying wire lines which incurs an extra cost. Use of an unlicensed band is also practical for cost efficient realization of broadband mobile communications; IEEE802.11 wireless LANs are the great majority. Considering the wireless multi-hop network based on wireless LAN access points, key challenges are related to its efficient relaying scheme. Throughput performance in multi-hop relaying originally decreases with the increase in hop count [17]. In addition, each node communicates based on a distributed coordination function (DCF), i.e., carrier sense multiple access with collision avoidance (CSMA/CA), so that the system is often dominated by packet collisions. It severely worsens the system capacity.

For tree topology based multi-hop networks, we previously proposed intermittent periodic transmit (IPT) protocol which achieves a high efficient packet forwarding in a simplified manner [18]. Its fundamental effectiveness has also been experimentally verified [19,20,21] and commercialization is ongoing [22]. Original IPT design was limited for uni-directional forwarding in a string-type topology. Its extensions for bi-directional traffic accommodation and 2-dimensional topologies have been developed. Bi-directional IPT (Bi-IPT) [23] is a simple modification where reverse link (uplink) transmission is also triggered by hearing of forward link (downlink) packet reception. Although Bi-IPT enables collision-free bi-directional duplex, its throughput improvement is limited due to the increase of packet delay especially in uplink. For 2-dimensional extension, path-reservation (PR-IPT) [24] and round robin packet scheduling (RR-IPT) [25] have been proposed. The former scheme forces an extended network allocation vector (NAV) to surrounding nodes on an IPT forwarding path. It prevents active nodes from packet transmission to awaiting nodes and hence it causes frequent retransmissions resulting in packet loss. In addition, RR-IPT with multi-channel packet forwarding [26] was found as an efficient solution keeping IPT’s advantages with accommodating up and downlink traffic. In [26], relay nodes have two wireless interfaces, each of which is assigned different channel delivering downlink and uplink traffic respectively. In this scheme, downlink traffic is exclusively assigned to the 1st channel and forwarded by RR-IPT. The 2nd channel is assigned uplink traffic with the use of the conventional CSMA/CA. However, its spectral efficiency cannot be maximized since uplink traffic is generally smaller than downlink; overall system capacity enhancement is limited. 

Meanwhile, there exists a lot of literature investigating multi-channel medium access control (MAC) protocols. Use of multiple channels can enhance the network capacity and it has been a challenge solving their efficient allocation as well as multi-channel hidden terminal problems in distributed/asynchronous manners. Handshakes in multiple channels [27] may cause gratuitous radiation interfering with other wireless nodes. Most of the solutions prepare a control channel [28,29,30] dedicated to channel determination. It may cause overhead called control channel bottleneck [29] and it wastes spectrum resources which cannot be used for data transmission. Assuming a single transceiver, channel switching incurs additional delay around several micro seconds [28,29,31]. Furthermore, the number of channels available is limited in practical situations. Equipping multiple transceiver [32] is proven to further enhance throughput performance by handling multiple traffic in parallel allowing additional installation cost. Soft frequency reuse approach has also been proposed in [33] which exploits the capture effect but necessitates a synchronization between multiple nodes with the help of periodic beacon, global positioning system (GPS) information or out-of-band solutions [34]. We should also consider interference from/to other wireless networks on the same frequency band, i.e., inter-system interference. Therefore, throughput performance should be maximized in limited spectrum resources.

IPT itself can resolve the hidden terminal problem by establishing the transmission period at the source node, hence, it does not require any handshakes for channel reservation nor control sequence. Periodical transmission approximately realizes synchronization among relay nodes. The most valuable feature of IPT is that it can maximize the throughput upon a single channel constraint. Key idea of our solution is to fully exploit it by introducing multi-channel and multi-interface approaches which can eliminate additional MAC protocols as well as dynamic channel switching but only requires an initial packet scheduling. Our goal is to maximize the system throughput of MWBN via minimized MAC protocol modifications. The major contribution of this paper is to propose a new multi-channel packet scheduling and suitable round robin based IPT forwarding. Computer simulation results exhibit that superior system throughput can be achieved without Bi-IPT on uplink traffic accommodation, especially in heavy offered load situation. The reminder of this paper is organized as follows. Section 2 summarizes related work and justifies the contribution of this paper. Section 3 reviews IPT and its extension, RR-IPT. Section 4 proposes RR-IPT based multi-channel packet scheduling and forwarding. Section 5 describes computer simulation methodologies and Section 6 presents results and discussions. Finally, Section 7 concludes this paper.

## 2. Related Work: Multi-Channel Multi-Interface Multi-Hop Networks

Channel assignment (CA) strategies for IEEE802.11 based multi-hop networks, wherein each node equips multi-interface (multi-transceivers), have been widely studied. The main problem is to improve the system throughput by assigning channels to wireless interfaces under the condition where the number of channels is larger than that of interfaces. There are significant proposals for CA protocols and their metrics are mainly classified as interference-aware, traffic-aware, congestion-aware, and delay, etc. [35]. Another viewpoint is from the operating layer: MAC layer alone, coordinated MAC and upper layers (i.e., cross layer), and their integrated approach (defined as common layer) [36]. In any case, each node should acquire arbitrary a priori information to perform CA in a distributed manner. Comprehensive analysis of multi-channel multi-interface multi-hop network based on distributed greedy CA algorithm was conducted in [37]. It proved the system throughput can be maximized even when the number of interfaces is less than that of available channels through extensive simulations using parameters such as the number of channels, wireless interfaces, interfering networks, and node densities. Reference [38] also evaluated the relationship between the number of sinks (gateways), channels, and interfaces. Results also verified the effectiveness of utilizing multi-interface with multi-channel rather than switching multi-channel in a single interface even when multiple sinks are deployed. Reference [39] proposed a distributed dynamic CA in which the node sounds the channel utilization status (considered as interference) of surrounding devices by use of beacon signals and manage them through a table. In addition to that, Reference [40] exploits information about channel usage notified from surrounding nodes and estimates interference amount. Each node then decides channels to use based on the game theory. Its detailed implementation is also disclosed in [41]. Above dynamic CA strategies require frequent channel/interface switching causing processing overhead. Hybrid approaches are also proposed in [42,43] in which fixed and switchable channels are prepared. Each node dynamically changes its own switchable channel for packet transmission according to the receiver’s channel fixedly assigned. Extended research was also done in [44] incorporating network coding technology. Our approach, being different to the above, can be placed as one of static (fixed) CA approach to completely eliminate overheads on interference presumption as well as channel switching. Doubling wireless interface/channel is to fully exploit the advantage of IPT which can maximize the forwarding efficiency in a single frequency channel.

## 3. Intermittent Periodic Transmit (IPT)

### 3.1. Principle and Fundamental Characteristics on String Tpology

**Figure 2:**
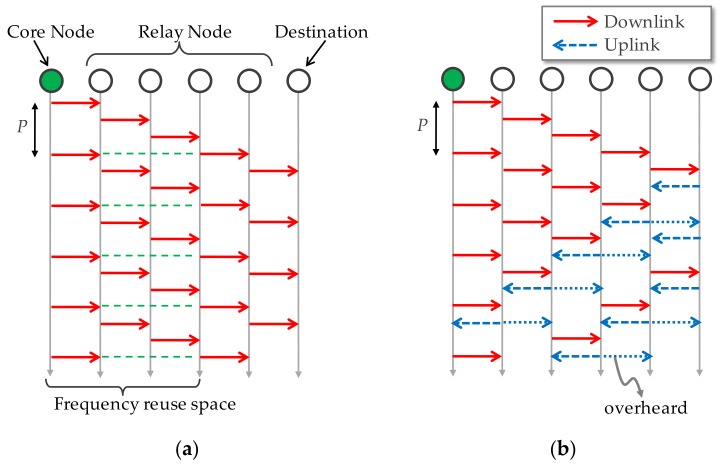
Intermittent Periodic Transmit (IPT). (**a**) Uni-directional IPT; (**b**) Bi-directional IPT.

**Figure 3:**
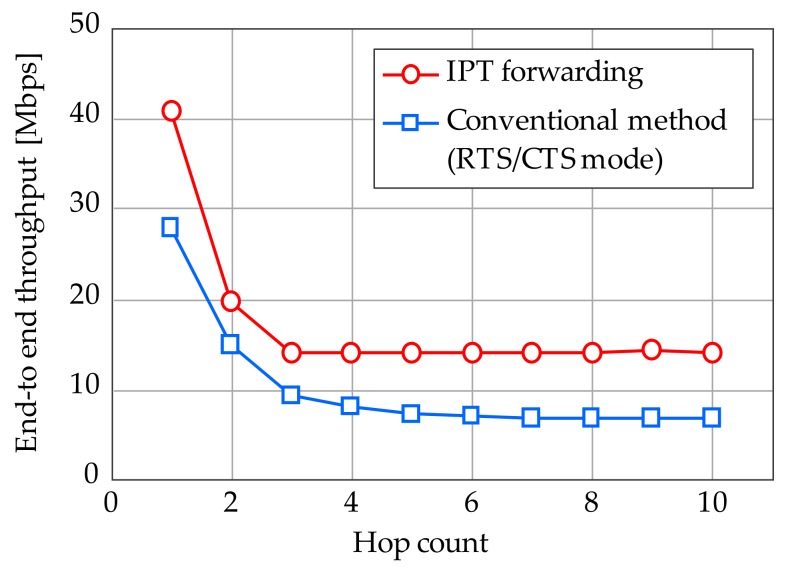
Basic throughput performance of IPT on string topology.

Figure 2 depicts the basic concept of IPT forwarding. As shown in Figure 2a, the source node periodically transmits packets with a constant interval, *P*, and relay nodes immediately forward their own packets after the reception of downlink packets, i.e., downlink packet flow is regarded as a polling signal. It is quite simple since the specific modification is required only in the core node. Interval *P* provides frequency reuse space (in this case, 3) and thus co-channel interference on the forwarding path, i.e., intra-path interference, can be avoided. It enables collision-free and highly efficient multi-hop relay while eliminating overheads such as request to send (RTS)/clear to send (CTS) handshakes and random back-off for CSMA/CA functions. Figure 3 plots basic throughput performance provided by IPT on the string topology compared to the RTS/CTS based CSMA/CA. This assumes transmission rate of 54 Mbps per hop and the signal radiation reaches for the adjacent nodes for simplicity. As shown, IPT forwarding can improve end-to-end throughput performance even while eliminating RTS/CTS function. Furthermore, throughput performance becomes independent of the hop count over 3 hops since the frequency reuse space of 3 is constantly kept.

### 3.2. Bi-Directional IPT (Bi-IPT)

Basic function of Bi-IPT is the same as IPT. It is a special case where relay nodes have uplink packets. Even if the sent packet is addressed to the upstream node, the opposite downstream node can receive (overhear) and it is regarded as a forward link packet reception, as shown in Figure 2b. Bi-IPT can also greatly reduce packet loss, however, it may increase packet delay especially in uplink traffic [23]. As mentioned above, this is because uplink traffic is carried after the downlink traffic reception. It should be noted that if there is no downlink packet to be sent, the node should transmit a control packet which has no data and it causes extra overhead.

### 3.3. Round Robin IPT (RR-IPT): Efficient Extention for Two-Dimensional Topology

**Figure 4:**
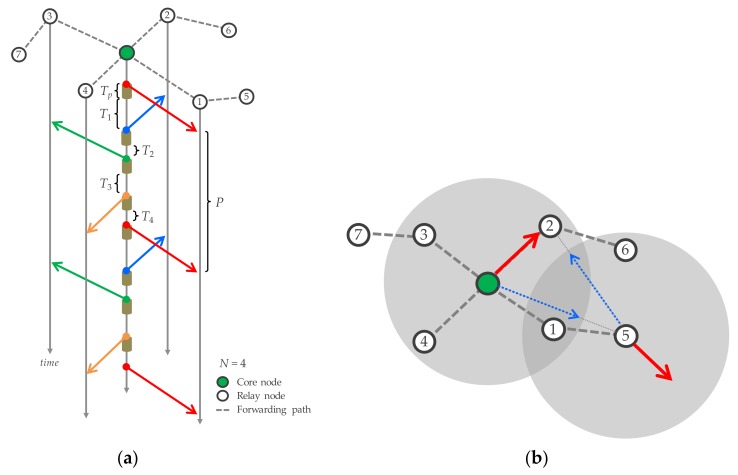
Round robin IPT (RR-IPT). (**a**) Transmission flow; (**b**) Frequency reuse on RR-IPT.

RR-IPT exploits the tree-topology of MWBN where forwarding paths are constructed around the core node. Role of the core node is predetermined depending on the deployment environment where wired Ethernet gateway is available. The tree topology is suitable for IPT since the core node acts as a control station. Wireless relay links on MWBN should be tolerant of an environmental fluctuation, so we employ minimum path loss routing [45,46] which can organize stable forwarding paths as well as ensure higher transmission rate. Its detailed routing algorithm is summarized in [46]. The core node intermittently transmits owing packets with predetermined interval and other nodes immediately relay received packets in a decode and forward (DF) manner. Figure 4 exemplifies detailed flow of RR-IPT. The core node sends out packets to surrounding nodes in successive order; consecutive transmission to the specific node is prohibited. With the conventional IPT in the string topology, the core node must wait for its transmission until the intermediate node in two (or more) hops away has finished transmitting. In other words, transmission period should be provided so that communication ranges (depicted as circles in the figure) cannot be overlapped.

In the tree topology, the transmission period can be set shorter than in the string topology by round robin packet transmission since the forwarding path (branch) is different as long as transmitted signals cannot interfere with each other, as illustrated in Figure 4b. Here, we consider the transmission period to be set for RR-IPT. Assuming packet transmission duration is *T_p_* for all nodes, let *N* and *T_i_* (*i* = 1, 2, …, *N*) denote the number of relay nodes next to the core node and the waiting time after the transmission to the *i*-th relay node, respectively, transmission period, *P*, for the specific branch can be expressed as follows.

(1) $$P=\;\sum_{i=1}^{N}\left(T_{p}+T_{i}\right)\;=NT_{p}+\sum_{i=1}^{N}T_{i}$$

Defining the required frequency reuse space as *M_i_*, a required transmission period for the *i*-th forwarding path, *S_i_*, is given by

(2) $$S_{i}=\;M_{i}\;T_{p}$$

In order to completely avoid intra-path interference, *P* ≥ *S_i_* is required for all *i*-th forwarding paths. From (1) and (2), following requirement can be derived;

(3) $$P-S_{i}=\;NT_{p}+\sum_{i=1}^{N}T_{i}-M_{i}T_{p}\;=(N-M_{i})\;T_{p}+\sum_{i=1}^{N}T_{i}\;\geq 0$$

It indicates that intra-path interference can be avoided when *N* ≥ *M_i_* even though *T_i_* is set to negligibly small value. In order to satisfy the above condition for arbitrary *i*-th forwarding path,

(4) $$N\geq \underset{i}{\max}(M_{i})$$

is required. Meanwhile, inter-path interference should also be considered in RR-IPT since IPT is performed to multiple forwarding paths unlike the string topology case. Although the most of intra-path and inter-path interference can be avoided by the use of transmission period, the former occasionally happens. Here we set minimum contention window (MinCW) as a small value but not zero and keep random back-off function activated so as to autonomously avoid inter-path interference while suppressing the overhead of CSMA/CA. In case a relay node has multiple forwarding paths, it also chooses subordinating relay nodes in round robin manner.

## 4. Proposed Multi-Channel Packet Scheduling and Forwarding

IPT is beneficial to achieve higher throughout especially in one way packet forwarding. In order to fully exploit this feature, we newly introduce a multi-channel packet scheduling, assigning one channel to downlink traffic and other to uplink and downlink traffic. Here we assume two channels and RR-IPT is applied to channel 1 (Ch.1) for downlink traffic forwarding. Generally, uplink traffic is lower than downlink and it is also small enough for the system capacity. This paper proposes the mixed accommodation of up/downlink traffic in channel 2 (Ch.2) for effective channel utilization. 

The core node has packet buffers prepared for respective channels and a threshold is established for Ch.1’s buffer. Downlink packets are preferentially assigned to Ch.1 and forwarded via RR-IPT. When the number of packets assigned to the Ch.1 buffer exceeds the predetermined threshold, thereafter newly originated packets are temporarily assigned to Ch.2. After the number of buffered packets for Ch.1 becomes less than the threshold, originated packets are assigned to Ch.1 again. Here assumes the threshold value as 100 packets and the number of downlink packets assigned to the Ch.2 buffer cannot exceed that of Ch.1 so as to avoid imbalance loading. Relay nodes utilize each channel independently; once the packet is assigned to the channel, it is forwarded to the destination node via the same channel (interface). This simple operation can eliminate overheads caused by the channel switching or dynamic CA per relay. This paper investigates following two types of packet forwarding schemes for Ch.2 where uplink and downlink traffic co-exist.

### 4.1. Mono-IPT

This paper newly investigates the applicability of conventional CSMA/CA in Ch.2, i.e., each node performs packet relay based on DCF manner. Overhead peculiar to the Bi-IPT’s interference avoidance function cannot be caused but that due to packet collisions and retransmissions must be allowed. 

### 4.2. Dual-IPT

In this scheme, uplink and downlink traffic assigned to Ch.2 are accommodated via Bi-IPT function. As stated in Section 3.1, its non-negligible overheads may limit forwarding efficiency in compensation for the packet loss reduction effect. Although employing directional antennas can alleviate such limitations [25], antenna switching overhead cannot also be negligible. This paper investigates this problem based on omnidirectional antenna condition from aspects of the practicality.

## 5. Computer Simulation

### 5.1. Wireless Transceiver

IEEE802.11a [47] specification is assumed as a radio transceiver in each node. Transmission power is assumed to be 20 dBm. Packet reception failure happens when signal-to-interference-plus-noise power ratio (SINR) level becomes lower than 10 dB. The combination of CSMA/CA with IPT may reduce forwarding efficiency because of the overhead due to the handshakes relating to the CSMA/CA operations. Even though such an unfavorable fact exists, this combination should remain for immunity to unexpected packet forwarding failures. Otherwise, nodes may not be able to exit from collision state forever. In order to reach a compromise between possibility of unexpected packet reception failures and the performance loss of IPT forwarding, MinCW is set shorter than the original value: 7 slots for nodes in IPT forwarding while the original 15 slots for ones in the normal mode. The maximum CW, on the other hand, is set to 1023 slots based on the specification of IEEE802.11a irrespective of forwarding modes.

Packet buffer is established as per adjoining relay node. Packet transmission for each buffer is managed in first-in first-out (FIFO) principle; when a relay node receives a packet to be forwarded, the packet is put into an adequate buffer at first and then it is sent out from the oldest packet in the buffer that was added most recently among all existing buffers. In case of RR-IPT, buffers are scheduled in round robin manner while it also follows FIFO manner in the conventional scheme. All buffers are assumed to be large enough so that the packet loss due to buffer overflow is not considered.

### 5.2. Evaluation Site

**Figure 5:**
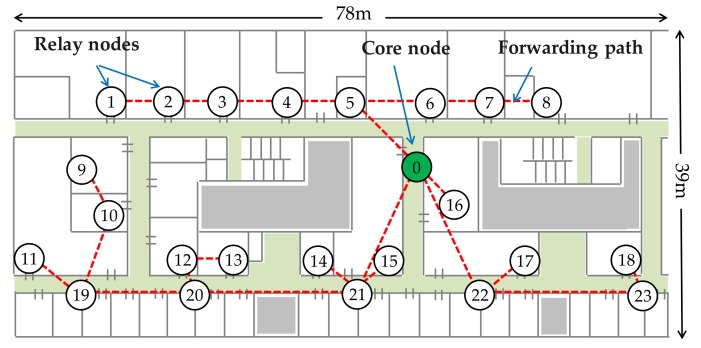
Node layout for evaluation.

Multi-hop networks are greatly influenced by radio propagation conditions. Since wireless LANs would be most operated in indoors, we choose a floor of department building of Kyushu University as a test site. Its floor plan is shown in Figure 5. Since typical communication range of wireless LAN modems is about 50 m and 100 m at most in indoor environments, a statistical modeling is inappropriate. In order to handle a complex interference situation as correctly as possible, we use a deterministic radio propagation model; a path loss coefficient is 2 until 5 m and is 3.5 beyond [48]. Penetration loss across per wall is 12 dB [49] and there is no short-term fading. Twenty-four wireless nodes are placed on the floor and a core node is on the center corridor. Figure 5 also indicates forwarding paths based on the minimum path loss routing [46] protocol. It is determined beforehand and fixed during simulations. Spectrum assigned to MWBN is assumed to be different from that for access links between user terminals and access points, thus interference between the relay network and the access network can be excluded. Our focus is placed on the relay network throughout the evaluation.

### 5.3. Traffic Model

Downlink traffic directed to user terminals that stay under relay nodes is all generated at the core node and forwarded to each relay node. Uplink traffic caused by user terminals is gathered at the relay nodes where users belong to and forwarded to the core node. The Poisson origination is employed as a traffic model. The number of data packets per session is randomly determined according to the log-normal distribution, the mean of which is 20 for downlink and 3 for uplink [50]. Assuming user datagram protocol (UDP), the ratio of the total offered load between downlink and uplink is set to 10:1 [51].

### 5.4. Transmission Period for IPT

When IPT is activated, the core node transmits owing packets according to the predetermined transmission period as summarized in Table 1. In this paper, it is manually determined while considering intra/inter-path interference. For instance, in the case that the destination node locates in 1 hop, intra/inter-path interference is improbable and thus the next buffered packet can be sent out in a transmission period of 0. Maximum hop count in the evaluation topology is *M_i_* = 4 and the number of adjacent nodes of the core node is also *N* = 4. It sufficiently satisfies the condition in (4) which ensures an efficient packet forwarding by RR-IPT. When RR-Bi-IPT is applied for Ch.2, we set a transmission period of 700 µs since the 9th node also transmits uplink packets and it causes inter-path interference with the 2nd node.

### 5.5. Evaluation Metrics

Simulative evaluation compares three metrics defined as follows.

- *System throughput* (bps): It is defined as the sum of throughputs for all sessions wherein packets are successfully delivered to a destination.
  (5) $$System\;throughput\;=\;\frac{1500\times 8N_{r}}{T_{sim}}$$
- *Average packet delay* (s): Average time period from the instant when a packet occurs at a source node to the instant when a destination node completes reception of the packet.
  (6) $$Average\;delay\;=\;\frac{1}{N_{r}}\sum_{i=1}^{N_{r}}D_{i}$$
- *Packet loss rate* (%): Ratio of the number of discarded packets to the total number of packets to be received.
  (7) $$Packet\;loss\;rate\;=\;\frac{N_{d}}{N_{r}+N_{d}}\times 100$$
  where *N_r_* denotes the number of packets successfully received by destination nodes. *D_i_* denotes the elapsed time period from the *i*-th packet’s occurrence at the source node to the reception completion at the destination node. *N_d_* denotes the number of discarded packets due to excess of the retry limit. Simulation is carried out for *T_sim_* = 240 s. This simulation period has been ensured to exhibit a good convergence.

We compare five schemes to be defined as follows. The same forwarding path is used for all schemes as shown in Figure 5.

**(A)**  ***DCH-Mono-IPT***—RR-IPT is applied to Ch.1 and it accommodates downlink traffic only. Uplink and partially downlink traffic are forwarded via Ch.2 using CSMA/CA.
**(B)**  ***DCH-Dual-IPT***—RR-IPT is applied to Ch.1 and it accommodates downlink traffic only. In Ch.2, uplink and downlink traffic are forwarded by RR-IPT in bi-directional manner (Bi-RR-IPT).
**(C)**  ***DCH-Conventional***—Conventional scheme is defined as that does not apply IPT but only CSMA/CA-based packet relaying. If a node succeeded packet transmission, it attempts to transmit a subsequent buffered packet after the random back-off period. Uplink and downlink traffic is also fairly assigned to each channel in FIFO manner, i.e., originated packets are alternately assigned to each transceiver so as to equalize offered load on each channel.
**(D)**  ***DCH-Previous***—Here we also compare the performance of RR-IPT-based scheme previously proposed in [26] where downlink traffic is assigned to Ch.1 and is forwarded via RR-IPT. Ch.2 accommodates only uplink traffic with the conventional CSMA/CA.
**(E)**  ***SCH-Conventional***—Uplink and downlink traffic are forwarded using the conventional CSMA/CA. It is a single channel version of (C) DCH-Conventional.

Above specifications are implemented on our original event-driven network simulator. Simulation proceeds per 1 µs step, and detailed workflows, e.g., random back-off, packet transmission with acknowledge (ACK) response, collision detection and retransmission, are modeled. Although analytical models have been proposed in [52,53], there are limitations on capable topologies: string or star types, the number of nodes, and the propagation ranges. As for the experiment, previous work validated the feasibility of IPT via prototype implementations [19,20,21]. This fact is applicable since our proposal separately treats each channel (transceiver).

## 6. Results and Discussion

**Figure 6:**
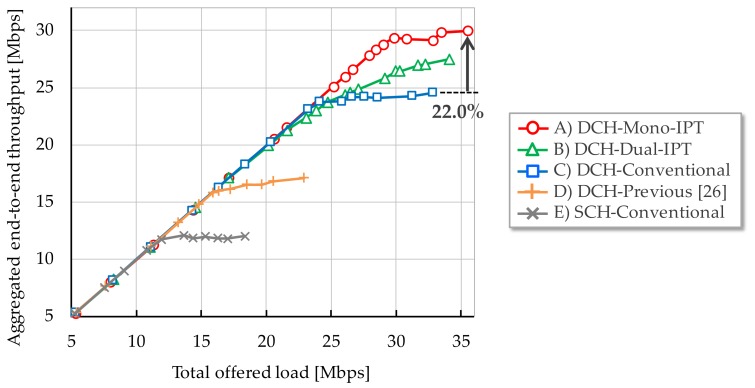
System throughput.

**Figure 7:**
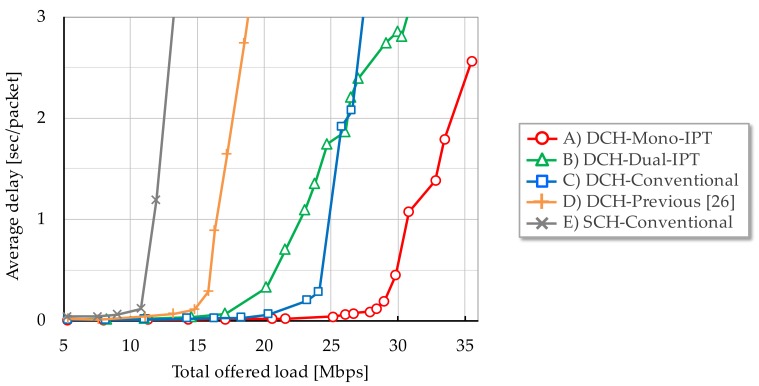
Average packet delay.

**Figure 8:**
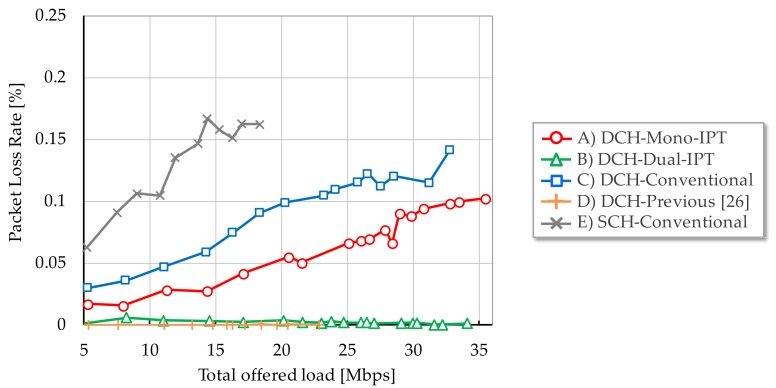
Packet loss rate.

Figure 6, Figure 7 and Figure 8 show each evaluation metric versus total offered load, respectively. From Figure 6, Mono-IPT achieved the best system throughput performance and its improvement is 22.0% compared to the conventional scheme with dual channel. Although the previous scheme outperforms the single channel case, it is still worse than other dual channel schemes. Obtained result implies that the proposed approach can fully exploit frequency resources. Throughput of Dual-IPT gradually increases as offered load but it cannot reach for Mono-IPT. What is worse, as the average delay performance shows in Figure 7, delay value of Dual-IPT shoot up in smaller offered load than the conventional scheme. This cannot be the best solution from the delay viewpoint. Mono-IPT also exhibits improved delay performance. Meanwhile, as shown in Figure 8, Dual-IPT achieves significantly reduced packet loss rate approaching to zero; it successfully obtains the feature of IPT interference avoidance function. Although Mono-IPT allows use of CSMA/CA in Ch.2 causing packet loss, it can reduce overall packet loss rate less than the conventional scheme by 30–50%. The following results present more detailed observations focusing on dual channel schemes (A)–(C).

**Figure 9:**
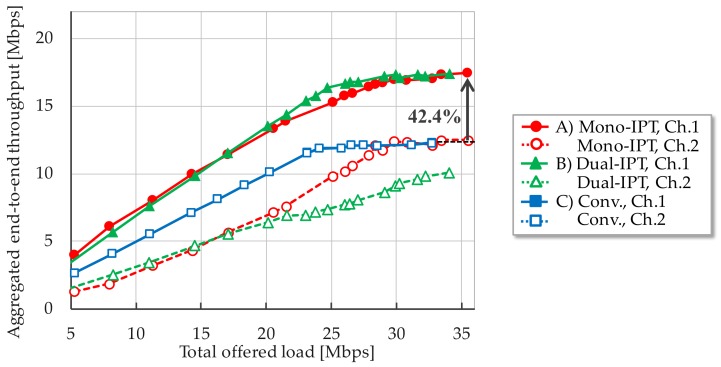
System throughput for each channel.

**Figure 10:**
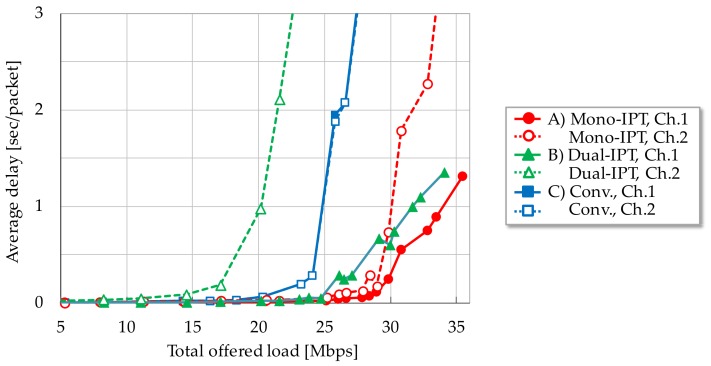
Average packet delay for each channel.

**Figure 11:**
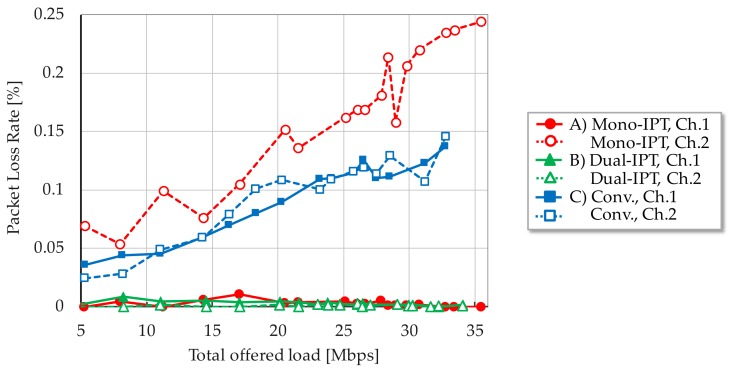
Packet loss rate for each channel.

Figure 9, Figure 10 and Figure 11 focused on system throughput, average packet delay and packet loss rate performances for each channel, respectively. As for Figure 9 and Figure 10, curves of the conventional scheme for each channel exhibited the same tendency, they coincide. With Mono-IPT, all metrics can be significantly improved in Ch.1 in which RR-IPT applied carrying only downlink traffic. Due to its enhanced capacity in Ch.1, downlink traffic load input to Ch.2 is reduced and the saturation point can be shifted to larger total offered load. It indicates that packets can be forwarded in reduced delay as far as input traffic reaches saturation point. Its tendency can also be confirmed from delay characteristics in Figure 10. As shown in Figure 11, packet loss rate of Mono-IPT in Ch.2 slightly increases compared to the conventional scheme. Since Ch.2 accommodates bi-directional traffic by CSMA/CA, the collision probability has been increased. RR-IPT can almost eliminate packet loss in Ch.1 and it contributes to improving the whole packet loss rate.

As a result, Mono-IPT is effective at improving overall system performance regarding both throughput and packet loss rate, notwithstanding employs CSMA/CA in partial channel. Dual-IPT faces limitation in Ch.2 wherein delay performance is deteriorated due to the non-negligible overhead of bi-directional traffic handling by IPT. It obstructs the fine feature of uni-directional IPT in Ch.1. Nevertheless, Dual-IPT can achieve significantly reduced packet loss rate and thus it is applicable in lower traffic situations. We can conclude that the solution is to switch RR-IPT based packet scheduling according to the offered traffic load. This can be performed by observing packet buffer filling level of the core node. This hybrid scheme can realize highly efficient MWBN in terms of both the communication quality and the system capacity. In the case of lower demand traffic, Dual-IPT is applied in order to realize an extremely reduced packet loss rate. When offered load grows, forwarding scheme is switched to Mono-IPT which can enhance the system capacity.

Although we employed simple IEEE802.11a standard, our approach can effectively work even in recent standards such as 802.11n/ac/ax. To assess the superiority provided by the modified function under the unified/fair constraint, MWBN based on 802.11a with CSMA/CA was sufficient as disclosed above. Of course recent extensions of 802.11 such as short guard interval (GI), aggregated MAC protocol data unit (A-MPDU), and multiple-input multiple-output (MIMO) functions can provide further enhanced throughput. The practical system has some limitations or uncertainty such as jitter, buffer upper limit, packet loss due to the channel conditions. Our future work should set up or introduce the above limitations into simulations via experiment.

## 7. Conclusions

This paper proposed a new multi-channel packet scheduling and forwarding scheme to fully exploit the advantage of RR-IPT. In the tree topology based dual channel network, we mainly compared two schemes; Dual-IPT applying RR-IPT to both channels and Mono-IPT where RR-IPT is applied to the specified single channel to accommodate only downlink traffic. Computer simulation revealed that Mono-IPT exhibited higher system throughout performance with lower delay, while Dual-IPT achieved completely reduced packet loss rate. The resultant solution is to switch these schemes depending on offered traffic load: observing packet buffer filling level on the core node. Hybrid RR-IPT based multi-channel packet forwarding can realize efficient MWBN ensuring both communication quality and the high capacity.

## Figures and Tables

**Table 1:** Simulation parameters.

Parameters	(A) DCH-Mono-IPT		(B) DCH-Dual-IPT		(C) DCH-Conv.		(D) DCH-Previous		(E) SCH-Conv.
	Ch.1	Ch.2	Ch.1	Ch.2	Ch.1	Ch.2	Ch.1	Ch.2	Ch.1
Wireless IF	IEEE 802.11a [47]								
Tx rate	54 Mbps								
Channel model	IEEE 802.11 TGn channel model D [48]								
	Wall penetration loss: 12 dB [49]								
Routing protocol	Minimum path loss routing [46]								
Traffic model	Poisson origination, Log-normal distribution								
	Avg. DL: 20 packets, UL: 3 packets [50]								
	Packet size: 1500 bytes,								
	Offered load ratio DL:UL=10:1 [51]								
Relay scheme	IPT	CSMA/CA	IPT	Bi-IPT	CSMA/CA		IPT	CSMA/CA	CSMA/CA
RTS/CTS	Off	On	Off	Off	On		Off	On	On
MinCW (slot)	7	15	7	7	15		7	15	15
Buffering	Round Robin	FIFO	Round Robin	Round Robin	FIFO		Round Robin	FIFO	FIFO
Traffic handling	DL	DL+UL	DL	DL+UL	DL+UL		DL	UL	DL+UL
Ch. switching threshold	100 packets	100 packets					100 packets	100 packets	
Tx period (μsec)	0, 100	0	0, 100	100, 700	0		0, 100	0	0

## References

[1] PicoCELA White Paper Cisco Visual Networking Index: Forecast and Methodology, 2016–2021. https://www.cisco.com/c/en/us/solutions/collateral/service-provider/visual-networking-index-vni/complete-white-paper-c11-481360.pdf.

[2] Atzori L., Iera A., Morabito G. (2010). The Internet of Things: A survey. Comput. Netw..

[3] Vermesan O., Friess P., Guillemin P., Gusmeroli S., Sundmaeker H., Bassi A., Jubert I.S., Mazura M., Harrison M., Eisenhauer M. (2011). Internet of Things strategic research roadmap. Internet Things‒Glob. Technol. Soc. Trends.

[4] Al-Fuqaha A., Guizani M., Mohammadi M., Aledhari M., Ayyash M. (2015). Internet of Things: A Survey on Enabling Technologies, Protocols, and Applications. IEEE Commun. Surv. Tutor..

[5] Ferreira da Silva F.J., Oliveira e Sá J. Internet-of-Things: Strategic research agenda evolution. Proceedings of the 11th Iberian Conference on Information Systems and Technologies (CISTI).

[6] Jain P., Hedman P., Zisimopoulos H. (2012). Machine type communications in 3GPP systems. IEEE Commun. Mag..

[7] Shariatmadari H., Ratasuk R., Iraji S., Laya A., Taleb T., Jäntti R., Ghosh A. (2015). Machine-type communications: Current status and future perspectives toward 5G systems. IEEE Commun. Mag..

[8] Bockelmann C., Pratas N., Nikopour H., Au K., Svensson T., Stefanovic C., Popovski P., Dekorsy A. (2016). Massive machine-type communications in 5g: Physical and MAC-layer solutions. IEEE Commun. Mag..

[9] Hoydis J., Kobayashi M., Debbah M. (2011). Green Small-Cell Networks. IEEE Veh. Technol. Mag..

[10] Hoadley J., Maveddat P. (2012). Enabling small cell deployment with HetNet. IEEE Wirel. Commun..

[11] Fehske A.J., Viering I., Voigt J., Sartori C., Redana S., Fettweis G.P. (2014). Small-Cell Self-Organizing Wireless Networks. Proc. IEEE.

[12] Chen D.C., Quek T.Q.S., Kountouris M. Wireless Backhaul in Small Cell Networks: Modelling and Analysis. Proceedings of the 2014 IEEE 79th Vehicular Technology Conference (VTC2014-Spring).

[13] Ge X., Cheng H., Guizani M., Han T. (2014). 5 G wireless backhaul networks: Challenges and research advances. IEEE Netw..

[14] Ge X., Pan L., Tu S., Chen H.H., Wang C.X. Wireless Backhaul Capacity of 5G Ultra-Dense Cellular Networks. Proceedings of the IEEE 84th Vehicular Technology Conference (VTC2016-Fall).

[15] Yamao Y., Suda H., Umeda N., Nakajima N. Radio Access Network Design Concept for the Fourth Generation Mobile Communication System. Proceedings of the IEEE 51st Vehicular Technology Conference (VTC2000-Spring).

[16] Pabst R., Walke B.H., Schultz D.C., Herhold P., Yanikomeroglu H., Mukherjee S., Viswanathan H., Lott M., Zirwas W., Dohler M. (2004). Relay-based deployment concepts for wireless and mobile broadband radio. IEEE Commun. Mag..

[17] Li J., Blake C., De Couto D.S.J., Lee H.I., Morris R. Capacity of ad hoc wireless networks. MobiCom ’01, Proceedings of the 7th Annual International Conference on Mobile Computing and Networking, Rome, Italy, 16–21 July 2001.

[18] Furukawa H. Hop Count Independent Throughput Realization by a New Wireless Multi-hop Relay. Proceedings of the 2004 IEEE 60th Vehicular Technology Conference (VTC2004-Fall).

[19] Higa Y., Furukawa H. (2007). Experimental evaluations of Wireless Multi-hop Networks associated with Intermittent Periodic Transmit. IEICE Trans. Commun..

[20] Jin G., Furukawa H. (2012). Automatic Transmission Period Setting for Intermittent Periodic Transmission in Wireless Backhaul System. IEICE Trans. Commun..

[21] Huang X., Muta O., Furukawa H. Performance evaluation of wireless multi-hop networks with directional antennas in an indoor radio propagation channel. Proceedings of the 2012 Japan-Egypt Conference on Electronics, Communications and Computers (JEC-ECC).

[22] PicoCELA White Paper A Wireless Backhaul Prototype with Capability of Over 10 Hops Relay. http://fukuoka.shoplog.jp/image/custom/picocela/kiji/whitePaper.pdf.

[23] Maruta K., Tohzaka Y., Higa Y., Furukawa H. Bidirectional Traffic Handlings in Wireless Multi-hop Networks Incorporating Intermittent Periodic Transmit and Packet Forwarding Path Reservation. Proceedings of the IEEE Asia Pacific Wireless Communications Symposium (APWCS2007).

[24] Tohzaka Y., Higa Y., Furukawa H. Evaluations of Wireless Multi-hop Network Incorporating Intermittent Periodic Transmit and Packet Forwarding Path Reservation. Proceedings of the IEEE 65th Vehicular Technology Conference (VTC2007-Spring).

[25] Mitsunaga K., Maruta K., Higa Y., Furukawa H. Application of directional antenna to wireless multi-hop network enabled by IPT for-warding. Proceedings of the 2nd International Conference on Signal Processing and Communication Systems (ICSPCS).

[26] Mohamed E.M., Kinoshita D., Mitsunaga K., Higa Y., Furukawa H. IEEE 802.11n based wireless backhaul enabled by Dual Channel IPT (DCH-IPT) relaying protocol. Proceedings of the International Congress on Ultra Modern Telecommunications and Control Systems.

[27] Muir A, Garcia-Luma-Aceves J.J. (1998). A channel access protocol for multi-hop wireless networks with multiple channels. Proceedings of the IEEE International Conference on Communications 1998 (ICC ’98).

[28] Luo T., Motani M., Srinivasan V. CAM-MAC: A Cooperative Asynchronous Multi-channel MAC Protocol for Ad Hoc Networks. Proceedings of the 3rd International Conference on Broadband Communications, Networks and Systems.

[29] Seo M., Kim Y., Ma J. Multi-channel MAC protocol for multi-hop wireless networks: Handling multi-channel hidden node problem using snooping. Proceedings of the IEEE Military Communications Conference (MILCOM 2008).

[30] Wang M., Wang M., Ci L., Xu Y. (2011). D-RDT: A Multi-channel MAC Protocol for Dense Multi-hop Wireless Sensor Networks. Procedia Eng..

[31] Papadopouli M., Porwal P. On-demand channel switching for multi-channel wireless MAC protocols. Proceedings of the 12th European Wireless Conference 2006—Enabling Technologies for Wireless Multimedia Communications (European Wireless).

[32] Iwabuchi M., Kishida A., Shintaku T., Onizawa T., Sakata T. Cooperative back-off control scheme for point-to-point simultaneous transmission using capture effect. Proceedings of the 2014 Asia-Pacific Microwave Conference (APMC ’14).

[33] So J., Vaidya N. Multi-channel mac for ad hoc networks: Handling multi-channel hidden terminals using a single transceiver. Proceedings of the 5th ACM international symposium on Mobile ad Hoc networking and computing (MobiHoc ’04).

[34] Xu C., Li G., Cheng W., Yang Z. Multi-transceiver multiple access (MTMA) for mobile wireless ad hoc networks. Proceedings of the IEEE International Conference on Communications 2005 (ICC ’05).

[35] Mogaibel H.A., Othman M., Subramaniam S., Hamid N.A.W.A. (2016). Review of channel assignment approaches in multi-radio multi-channel wireless mesh network. J. Netw. Comput. Appl..

[36] Kaabi F., Ghannay S., Filali F. (2010). Channel Allocation and Routing in Wireless Mesh Networks: A survey and qualitative comparison between schemes. Int. J. Wirel. Mob. Netw..

[37] Merlin S., Vaidya N., Zorzi M. Resource Allocation in Multi-Radio Multi-Channel Multi-Hop Wireless Networks. Proceedings of the IEEE the 27th Conference on Computer Communications (INFOCOM 2008).

[38] Campbell C.A., Khan S., Singh D., Loo K.K. (2011). Multi-Channel Multi-Radio Using 802.11 Based Media Access for Sink Nodes in Wireless Sensor Networks. Sensors.

[39] Jembre Y.Z., Li Z., Hiroo S., Komuro N., Choi Y.J. Channel assignment for multi-interface multi-hop wireless networks. Proceedings of the International Conference on Information and Communication Technology Convergence (ICTC).

[40] Amiri-Nezhad M., Cerdà-Alabern L. Adaptive Channel Assignment for Wireless Mesh Networks using Game Theory. Proceedings of the IEEE Eighth International Conference on Mobile Ad-Hoc and Sensor Systems (MASS 2011).

[41] Amiri-Nezhad M., Guerrero-Zapata M., Bellalta B., Cerdà-Alabern L. (2014). Simulation of multi-radio multi-channel 802.11-based mesh networks in ns-3. EURASIP J. Wirel. Commun. Netw..

[42] Kyasanur P., Vaidya N.H. Routing and interface assignment in multi-channel multi-interface wireless networks. Proceedings of the IEEE Wireless Communications and Networking Conference (WCNC2005).

[43] Li C.Y., Jeng A.K., Jan R.H. (2007). A MAC Protocol for Multi-Channel Multi-Interface Wireless Mesh Network using Hybrid Channel Assignment Scheme. J. Inf. Sci. Eng..

[44] Ning Z., Song Q., Guo L., Kong X. (2015). A novel adaptive spectrum allocation scheme for multi-channel multi-radio wireless mesh networks. J. Netw. Comput. Appl..

[45] Jin G., Furukawa H. Stable Routing Protocol Based on Successive Average of RSSI between Relay Nodes. Proceedings of the IEEE Asia Pacific Wireless Communications Symposium (APWCS2010).

[46] Suetsugu T., Furukawa H. (2017). Routing Algorithm for Wireless Multi-hop Networks with Tree Topology for Smart Meter Systems. Int. J. Eng. Technol..

[47] IEEE Std 802.11a (1999). Supplement to IEEE Standard for Information Technology—Telecommunications and Information Exchange between Systems—Local and Metropolitan Area Networks—Specific Requirements—Part 11: Wireless LAN Medium Access Control (MAC) and Physical Layer (PHY) specifications: High-Speed Physical Layer in the 5 GHz Band.

[48] Erceg V., Schumacher L., Kyritsi P., Molisch A., Baum D.S., Gorokhov A.Y., Oestges C., Li Q., Yu K., Tal N. (2004). TGn Channel Models. https://mentor.ieee.org/802.11/dcn/03/11-03-0940-04-000n-tgn-channel-models.doc.

[49] P.1238: Propagation Data and Prediction Models for the Planning of Indoor Radio Communication Systems and Radio Local Area Networks in the Frequency Range 300 MHz to 100 GHz. https://www.itu.int/rec/R-REC-P.1238-9-201706-I/en.

[50] Abrams M., Williams S., Abdulla G., Patel S., Ribler R., Fox E.A. Multimedia traffic analysis using Chitra95. Proceedings of the Third ACM International Conference on Multimedia (Multimedia ’95).

[51] Cho K., Fukuda K., Esaki H., Kato A. Observing Slow Crustal Movement in Residential User Traffic. Proceedings of the 2008 ACM CoNEXT Conference.

[52] Sanada K., Sekiya H. (2017). Bottom-up analysis concept for throughput and delay analyses of wireless multi-hop networks. Nonlinear Theory Its Appl. IEICE.

[53] Hussein O., Sadek N.M., Elnoubi S., Rizk M.R.M. (2014). Analytical Model of Multi-hop IEEE 802.15.4 with Unslotted CA/CSMA. Int. J. Comput. Commun. Eng..

